# Control of PD-L1 expression by miR-140/142/340/383 and oncogenic activation of the OCT4–miR-18a pathway in cervical cancer

**DOI:** 10.1038/s41388-018-0347-4

**Published:** 2018-05-31

**Authors:** Peixin Dong, Ying Xiong, Jiehai Yu, Lin Chen, Tang Tao, Song Yi, Sharon J. B. Hanley, Junming Yue, Hidemichi Watari, Noriaki Sakuragi

**Affiliations:** 1 Department of Women’s Health Educational System, Hokkaido University School of Medicine, Hokkaido University, Sapporo, 0608638 Japan; 2 Department of Obstetrics and Gynecology, Hokkaido University School of Medicine, Hokkaido University, Sapporo, 0608638 Japan; 3 Department of Gynecology, State Key Laboratory of Oncology in South China, Sun Yat-Sen University Cancer Center, 510060 Guangzhou, China; 4 Faculty of Medicine, Department of Obstetrics & Gynaecology, The Chinese University of Hong Kong, Shatin, New Territories, Hong Kong, China; 5 Department of Pathology and Laboratory Medicine, University of Tennessee Health Science Center, Memphis, TN 38163 USA; 6 Center for Cancer Research, University of Tennessee Health Science Center, Memphis, TN 38163 USA

**Keywords:** Cervical cancer, Immunosurveillance

## Abstract

PD-L1, a key inhibitory immune receptor, has crucial functions in cancer immune evasion, but whether PD-L1 promotes the malignant properties of cervical cancer (CC) cells and the mechanism by which PD-L1 is regulated in CC remains unclear. We report that PD-L1 is overexpressed in CC, and shRNA-mediated PD-L1 depletion suppresses the proliferation, invasion, and tumorigenesis of CC cells. Loss of miR-140/142/340/383 contributes to PD-L1 upregulation. miR-18a enhances PD-L1 levels by targeting *PTEN*, *WNK2* (ERK1/2 pathway inhibitor), and *SOX6* (Wnt/β-catenin pathway inhibitor and p53 pathway activator) to activate the PI3K/AKT, MEK/ERK, and Wnt/β-catenin pathways and inhibit the p53 pathway, and miR-18a also directly suppresses the expression of the tumor suppressors BTG3 and RBSP3 (CTDSPL). miR-18a overexpression in CC cells is triggered by OCT4 overexpression. Our data implicate PD-L1 as a novel oncoprotein and indicate that miR-140/142/340/383 and miR-18a are key upstream regulators of PD-L1 and potential targets for CC treatment.

## Introduction

Cervical cancer (CC) is the fourth most common malignancy in women and the fourth leading cause of cancer-related deaths among women worldwide [1, 2]. Tumors suppress the host immune system by upregulating programmed death ligand 1 (PD-L1) that binds to programmed death-1 on T cells, resulting in inhibitory checkpoint signaling that inhibits T cell expansion and function [3–5]. Overexpression of PD-L1 has been found in human cancers, including CC and pancreatic cancer [6–8]. In addition to mediating T cell suppression, recent studies have shown the critical roles of PD-L1 in promoting cancer cell growth and invasion [9–11]. However, the exact biological function of PD-L1 in CC remains unclear.

EGFR mutation, PTEN deletion, PI3K or AKT mutations, aberrant JAK/STAT signaling, and Wnt/β-catenin signaling activation can induce PD-L1 expression [12–16]. MicroRNAs (miRNAs) are critical regulators of cancer metastasis [17–19]. miR-513 and miR-570 target PD-L1, while p53 indirectly inhibits PD-L1 levels by inducing miR-34a expression [20–22]. The miRNAs that have the capacity to modulate PD-L1 expression in CC remains unknown.

We hypothesize that PD-L1 not only promotes tumor immune escape, it also enhances the malignant properties of CC cells. In the present study, we found that PD-L1 is overexpressed in CC and is an important promoter of CC cell proliferation and invasion. We also identify two novel mechanisms, including a miR-140/142/340/383–PD-L1 axis and an OCT4-miR-18a-PTEN/WNK2/SOX6 axis, that are responsible for the upregulation of oncoprotein PD-L1 in CC, suggesting that targeting PD-L1 by introducing miR-140/miR-142/miR-340/miR-383 or silencing of miR-18a might represent a therapeutic option to repress the metastatic phenotypes of CC cells and simultaneously reverse the immunosuppressive CC microenvironment.

## Results

### PD-L1 is aberrantly expressed in primary CC samples and CC cell lines

**Fig. 1:**
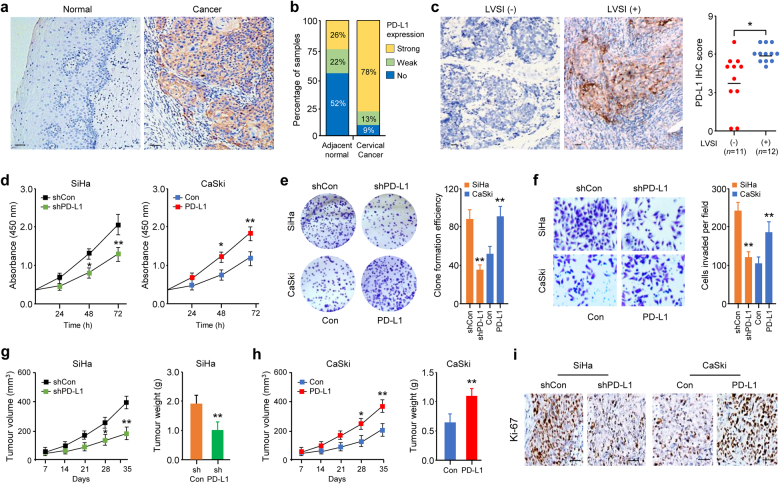
PD-L1 is upregulated in cervical cancer patients and is required for tumor growth and invasion. **a** Representative immunohistochemistry (IHC) image of PD-L1 in 23 paired human cervical cancer (CC) and adjacent normal cervical specimens (scale bar: 50 µm). **b** Percentage of cases that exhibit different PD-L1 protein expression levels (no staining, weak, or strong) in 23 paired human CC tissues and adjacent normal tissues (*P* < 0.01). **c** Representative IHC images of PD-L1 (left panel) and scatter plots showing the IHC scores for PD-L1 protein in patients with or without lymphovascular space invasion (LVSI, right panel). Scale bar: 50 µm. **d** Growth curves of PD-L1-knockdown SiHa cells and CaSki cells with ectopic PD-L1 expression (*n* = 6). **e** Representative images (left panel) and data quantification (right panel) of colony formation of PD-L1-knockdown SiHa cells and CaSki cells with ectopic PD-L1 expression (*n* = 6). **f** Representative images (left panel) and data quantification (right panel) of Matrigel invasion assays of PD-L1-knockdown SiHa cells and CaSki cells with ectopic PD-L1 expression (*n* = 6). **g**, **h** Growth curves and quantification of weight of tumors that developed in nude mice injected subcutaneously with PD-L1-knockdown SiHa cells (**g**) or PD-L1-overexpressing CaSki cells (**h**) (*n* = 8 per group). **i** Representative IHC images of Ki-67 in tumors from the mice described in **g**, **h**. Scale bar: 50 µm. *P < 0.05, **P < 0.01

We evaluated PD-L1 expression using immunohistochemical (IHC) analysis of 23 primary CC and paired adjacent normal tissue specimens. A strong PD-L1 staining was observed in CC samples (Fig. 1a). 78% of the cancer tissues displayed strong PD-L1 expression, whereas most adjacent normal samples (74%) showed no or weak PD-L1 expression (*P* < 0.01, Fig. 1b). The staining intensity of PD-L1 in CC was significantly higher than that in adjacent normal samples (2.61 ± 0.19 vs. 1.0 ± 0.14, *P* < 0.01). Higher PD-L1 expression levels were associated with a higher incidence of lymphovascular space invasion (Fig. 1c).

We next assessed PD-L1 expression in a panel of CC cell lines and the immortalized human endometrial epithelial EM cell line using immunoblotting. Compared with EM cells that do not have significant PD-L1 expression, CC cell lines overexpressed PD-L1 (Supplementary Fig. 1a). The highest level of PD-L1 was observed in SiHa cells, which have a high capacity to invade in a Matrigel cell invasion assays [23] (Supplementary Fig. 1a), suggesting that PD-L1 may function as an oncoprotein in CC.

### PD-L1 promotes CC cell proliferation and invasion

To examine the contribution of endogenous PD-L1 to the proliferative and invasive phenotype of CC cells, we performed PD-L1 loss-of-function and gain-of-function experiments in CC cell lines (Supplementary Fig. S1a). Short hairpin RNA-mediated knockdown of PD-L1 decreased SiHa cell proliferation and invasion, and stable overexpression of PD-L1 in CaSki cells promoted their proliferation and invasion (Fig. 1d–f).

We next explored the biological function of PD-L1 in CC tumorigenesis in vivo by injecting PD-L1-knockdown SiHa cells or PD-L1-overexpressing CaSki cells into nude mice. PD-L1-knockdown SiHa cells generated smaller tumors, and PD-L1-overexpressing CaSki cells formed larger tumors than control cells (Fig. 1g, h). PD-L1-depleted SiHa tumors exhibited downregulation of Ki-67, but PD-L1-overexpressing CaSki tumors showed upregulation of Ki-67 (Fig. 1i). To examine whether the cytoplasmic domain of PD-L1 plays a critical role in promoting CC cell invasion and proliferation, we overexpressed either wild-type (WT) PD-L1 or cytoplasmic domain-truncated PD-L1 and observed that CaSki cells overexpressing WT PD-L1, but not cytoplasmic domain-truncated PD-L1, exhibited significantly higher proliferation and invasion than the control cells (Supplementary Fig. S1a), suggesting that PD-L1 induces CC cell proliferation and invasion.

### Identification of miRNAs that regulate PD-L1 expression

To identify miRNAs that suppress CC invasion and metastasis, we isolated highly invasive subpopulations of SiHa cells using Matrigel Invasion Chambers [24]. In contrast to parental SiHa cells, the majority of invasive SiHa cells exhibited a spindle-shaped and mesenchymal-like morphology (Supplementary Fig. S2a). Compared with the parental cells, the invasive SiHa sublines showed enhanced invasion abilities (Supplementary Fig. S2a).

Then, a miRNA microarray assay was performed, and the expression profiles of invasive SiHa cells and their parental cells were determined (Fig. S2b; Supplementary Table S1). We merged the obtained list of downregulated miRNAs in highly invasive SiHa cells obtained by microarray assay with miRNAs predicted by an in silico analysis, and identified miR-140/142/340/383 as potential regulators of PD-L1 in CC cells (Supplementary Fig. S1c). Similarly, we defined miR-18a, which was upregulated in highly invasive SiHa cells (Supplementary Figs. S1c, S2b), and may act as the suppressor of PTEN, an upstream repressor of PD-L1 [3].

Real-time RT-PCR (qRT-PCR) assay confirmed that miR-140/142/340/383 were significantly downregulated, whereas miR-18a was significantly overexpressed in CC cell lines compared with normal EM cells (Supplementary Fig. S1d). qRT-PCR assay validated the lower miR-140/142/340/383 expression or higher miR-18a expression in 60 primary CC tissues relative to matched adjacent normal tissues (Supplementary Fig. S2c). In CC samples, *PD-L1* expression was positively correlated with miR-18a expression, but inversely correlated with miR-140/142/340/383 expression (Supplementary Fig. S2d). CC patients with higher miR-18a expression or lower miR-140/142/340/383 expression had a shorter survival time (Supplementary Fig. S2e).

We tested whether *PD-L1* mRNA expression is regulated by these identified miRNAs. Transient transfection of the miR-140/142/340/383 mimic or anti-miR-18a inhibitor reduced PD-L1 expression in SiHa cells. Conversely, transfection of the miR-18a mimic or anti-miR-140/142/340/383 inhibitors increased PD-L1 expression in CaSki cells (Supplementary Fig. S1e, f).

### PD-L1 is directly repressed by the miR-140/142/340/383 tumor suppressors

**Fig. 2:**
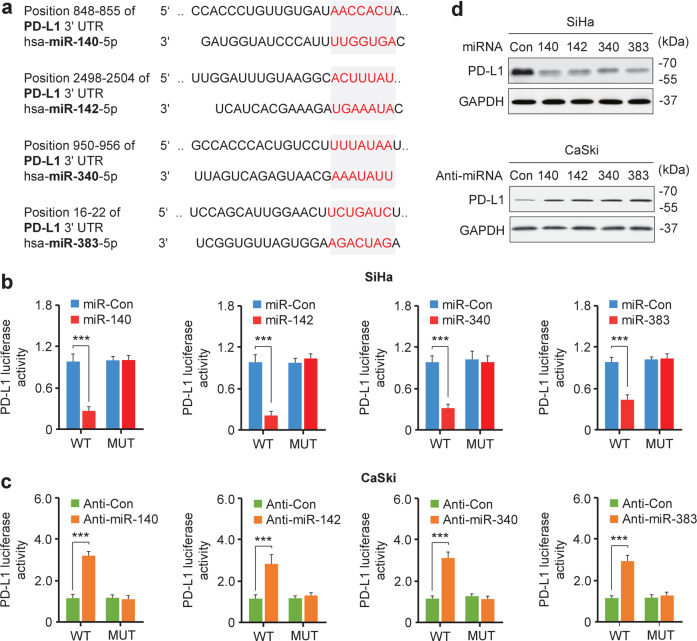
PD-L1 is directly repressed by the miR-140/142/340/383 tumor suppressors. **a** Predicted miR-140, miR-142, miR-340, and miR-383 binding sites in the 3′-UTR of *PD-L1*. **b**, **c** Luciferase reporter assays of SiHa cells co-transfected with a reporter construct containing the wild-type (WT) or mutant (MU) 3′-UTR of PD-L1 and the indicated miRNA mimics or their controls (**b**) and of CaSki cells co-transfected with a reporter construct containing the WT or MU 3′-UTR of PD-L1 and the indicated anti-miRNA inhibitors or their negative controls (**c**) (*n* = 4). **d** Western blot analysis of PD-L1 in SiHa (upper panel) and CaSki (lower panel) cells transfected as indicated (*n* = 3). ***P < 0.001

We performed the luciferase reporter assays by co-transfecting CC cells with a luciferase reporter plasmid fused to WT *PD-L1* 3′-UTR or mutant *PD-L1* 3′-UTR harboring mutations in the putative miR-140/142/340/383 binding sites, together with miR-140/142/340/383 mimics or anti-miR-140/142/340/383 inhibitors. The luciferase activity of the WT *PD-L1* reporter was reduced by miR-140/142/340/383 overexpression, but induced by anti-miR-140/142/340/383 inhibitors in CC cells (Fig. 2a–c). Mutation of the binding sites abolished the effects of miR-140/142/340/383 on the luciferase activity (Fig. 2a–c). miR-140/142/340/383 overexpression decreased PD-L1 protein expression, and knockdown of these miRNAs increased the PD-L1 protein levels in CC cells (Fig. 2d), indicating that miR-140/142/340/383 directly target the *PD-L1* 3′-UTR.

Ectopic PD-L1 expression in SiHa cells overcame the suppressive effects of miR-140/142/340/383 mimics on cell proliferation and invasion (Supplementary Fig. S3). SiRNA-mediated PD-L1 silencing in CaSki cells suppressed the proliferation and invasion, which were promoted by anti-miR-140/142/340/383 inhibitors (Supplementary Fig. S3), suggesting that the PD-L1 mediates the suppressive effects of miR-140/142/340/383.

### miR-18a promotes CC cell proliferation and invasion

We investigated the biological role of miR-18a in CC cells, and found that downregulating miR-18a levels in SiHa cells decreased proliferation and invasion, whereas overexpressing miR-18a in ME-180 and CaSki cells increased proliferation and invasion (Supplementary Fig. S4a, b). To further explore the cellular function of miR-18a, we generated miR-18a-knockout SiHa cell lines using the CRISPR/Cas9 gene editing system by transfecting the lenti-CRISPR/Cas9-miR-18a vectors containing the individual sgRNAs into SiHa cells (Supplementary Fig. S4c, d). The T7EN1 assay [25] showed the cleavage bands, suggesting that CRISPR/Cas9 induced mutations at the *miR-18a* locus (Supplementary Fig. S4e). Among the miR-18a-knockout clones, we identified two clones that carried a 4-bp deletion or a 10-bp deletion (Supplementary Fig. S4f). Deletion of 4 nucleotides significantly reduced and deletion of 10 nucleotides dramatically reduced (by more than 90%) the expression of mature miR-18a in SiHa cells (Supplementary Fig. S4g). miR-18a knockout significantly repressed CC cell proliferation and invasion (Supplementary Fig. S4h, i). To determine the effects of PD-L1 disruption in vivo, nude mice were subcutaneously injected with SiHa cells with PD-L1-knockout or control cells. Mice injected with PD-L1-knockout cells developed smaller subcutaneous tumors than those injected with control cells (Supplementary Fig. S4j, k), indicating that miR-18a promotes a metastatic phenotype in CC cells.

### miR-18a enhances PD-L1 expression by repressing PTEN and WNK2

**Fig. 3:**
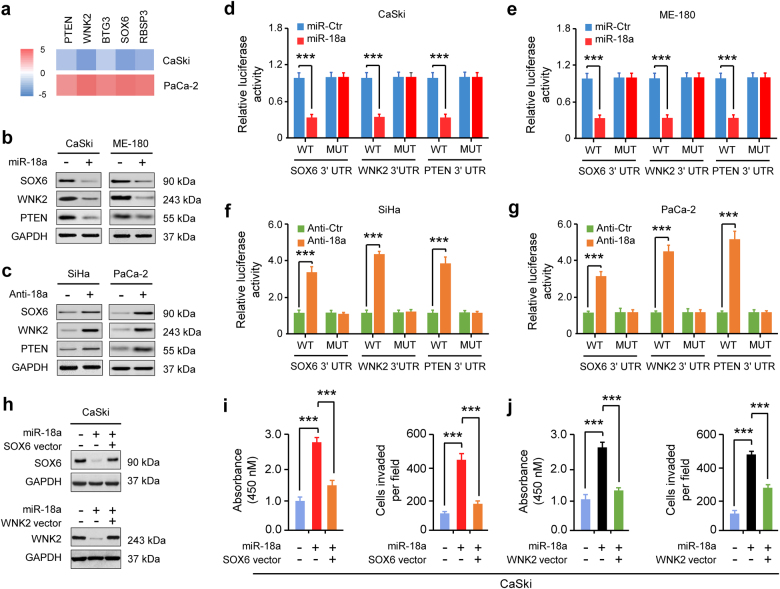
miR-18a directly targets the PTEN, SOX6, and WNK2 tumor suppressors. **a** Heat map of qRT-PCR data showing the expression of five genes (*PTEN*, *WNK2*, *BTG3*, *SOX6*, and *RBSP3*) that were downregulated by miR-18a overexpression in CaSki cells, but were upregulated by miR-18a knockdown in PaCa-2 cells (*n* = 3). Red: upregulation of mRNA; green: downregulation of mRNA. **b**, **c** Western blotting analysis of SOX6, WNK2, and PTEN in CaSki and ME-180 cells transfected with the miR-18a mimic or its negative control (**b**) and in SiHa and PaCa-2 cells transfected with the anti-miR-18a inhibitor or its negative control (**c**) (*n* = 3). **d**–**g** Luciferase reporter assays of CaSki (**d**), ME-180 (**e**), SiHa (**f**), and PaCa-2 (**g**) cells co-transfected with wild-type (WT) or mutant (MU) *SOX6*, *WNK2*, and *PTEN* 3′-UTRs and the miR-18a mimic, anti-miR-18a inhibitor or the appropriate negative control as indicated (*n* = 4). **h** Western blotting analysis of SOX6 and WNK2 in CaSki cells co-transfected with the miR-18a mimic or its negative control and the SOX6, WNK2 expression vector or control (*n* = 3). **i**, **j** Cell proliferation assays (**i**) and Matrigel invasion assays (**j**) of CaSki cells transfected with the miR-18a mimic or its negative control with or without the SOX6 or WNK2 expression vector (*n* = 6). ***P < 0.001

By combining target prediction and qRT-PCR profiling, we found gene transcripts that were downregulated by miR-18a overexpression in CaSki cells and that were upregulated by miR-18a knockdown in PaCa-2 cells, and identified five candidate genes (*PTEN*, *WNK2*, *SOX6*, *BTG3*, and *RBSP3*) that we defined as candidate targets of miR-18a (Fig. 3a; Supplementary Figs. S1d, S5).

Since *PTEN*, *WNK2*, *SOX6*, *BTG3*, and *RBSP3* have putative miR-18a-binding sites in their 3′-UTRs (Supplementary Fig. S6a) and act as tumor suppressors in CC [18, 26–28], we hypothesized that miR-18a enhances PD-L1 expression via targeting these candidate targets.

PTEN, SOX6, WNK2, BTG3, and RBSP3 protein expression was decreased in miR-18a-overexpressing CC cells but was increased in miR-18a-silenced cancer cells (Fig. 3b,c; Supplementary Fig. S6b). Luciferase reporter assays showed that reporter activities driven by the 3′-UTRs of *PTEN*, *SOX6*, *WNK2*, *BTG3*, and *RBSP3* were attenuated by miR-18a overexpression but elevated by miR-18a silencing. Mutations of the 3′-UTRs of these genes abrogated the effects of miR-18a, suggesting that they are direct targets of miR-18a (Fig. 3d–g; Supplementary Fig. S6c, d). Moreover, individual ectopic expression of SOX6, WNK2, BTG3, and RBSP3 significantly inhibited miR-18a-induced CC cell proliferation and invasion (Fig. 3h–j; Supplementary Figs. S6e–h, S7a–c), indicating that PTEN, WNK2, SOX6, BTG3, and RBSP3 are functional and direct targets of miR-18a in CC cells.

Consistent with the inhibitory effect of PTEN on PD-L1 expression [12], western blotting analysis showed upregulation of PTEN and downregulation of p-AKT and PD-L1 in SiHa cells with CRISPR/Cas9-mediated depletion of miR-18a (Supplementary Fig. S4g), supporting the notion that miR-18a silencing activates the PI3K/AKT pathway by targeting PTEN, and thereby upregulating PD-L1 expression.

WNK2 negatively regulates the MEK/ERK pathway in CC cells [26]. We explored the role of the WNK2/MEK/ERK pathway in regulating miR-18a-induced PD-L1 expression. Overexpression of miR-18a increased ERK1/2 phosphorylation and upregulated PD-L1 expression; however, forced WNK2 expression blocked miR-18a-induced MEK/ERK pathway activation and reduced PD-L1 expression (Supplementary Fig. S7d), suggesting that miR-18a induces PD-L1 levels by targeting WNK2 to activate the MEK/ERK pathway. To further support these findings, we treated CC cells with the MEK inhibitor PD0325901. Inhibition of the MEK/ERK pathway with PD0325901 resulted in decreased PD-L1 expression and attenuated miR-18a-enhanced CC cell proliferation and invasion (Supplementary Fig. S7e–g). These data suggest that miR-18a-mediated PTEN and WNK2 inhibition and subsequent activation of the PI3K/AKT and MEK/ERK pathways contributes to PD-L1 upregulation in CC cells.

### miR-18a increases PD-L1 levels by targeting SOX6 to activate the Wnt/β-catenin pathway and inactivate p53 signaling

**Fig. 4:**
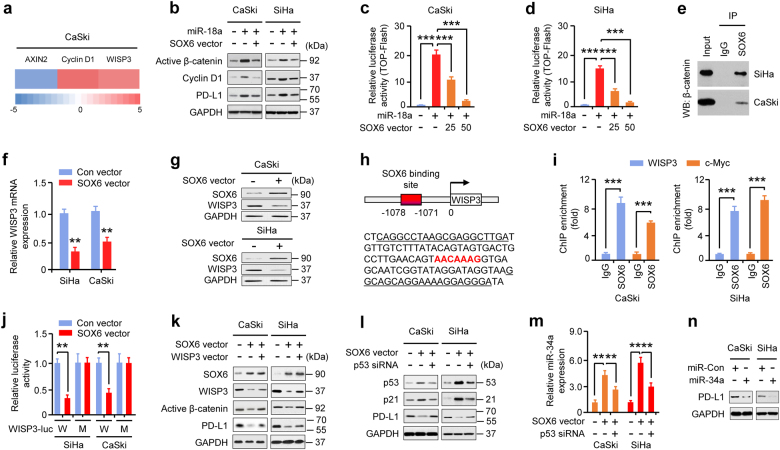
miR-18a increases PD-L1 levels by repressing SOX6 to activate the Wnt/β-catenin pathway and inactivate p53 signaling. **a** Heat map of qRT-PCR data showing the expression of Wnt signaling pathway-related genes in CaSki cells transfected with the miR-18a mimic or its negative control. Red: upregulation of mRNA; green: downregulation of mRNA. **b** Western blotting analysis of active β-catenin, cyclin D1, and PD-L1 in CaSki and SiHa cells transfected with the miR-18a mimic or its negative control with or without the SOX6 expression vector (*n* = 3). **c**, **d** TOP/FOP-Flash reporter assays of CaSki (**c**) and SiHa (**d**) cells co-transfected with the miR-18a mimic or its negative control and the SOX6 expression vector or control (*n* = 4). **e** Co-immunoprecipitation assays reveal associations between endogenous SOX6 and β-catenin in CC cells. Cell lysates are labeled as input. IgG was used as the control (*n* = 3). **f**, **g** qRT-PCR (**f**) and western blotting (**g**) of WISP3 expression in CaSki and SiHa cells transfected with the SOX6 expression vector or control vector (*n* = 3). **h** Schematic representation of the human *WISP3* gene promoter. The predicted SOX6 binding site is shown in red, and the primer sequences for ChIP-qPCR assays are underlined. **i** SOX6 occupancy at its potential binding site in the *WISP3* gene promoter was analyzed using ChIP-qPCR assays. c-Myc, a known SOX6 target, was used as a positive control (*n* = 3). **j** CaSki and SiHa cells were transfected with the wild-type (WT) or mutant (MU) *WISP3*-promoter luciferase plasmid with or without the SOX6 expression vector. Relative luciferase activity was assessed (*n* = 4). **k** Western blot analysis of SOX6, WISP3, active β-catenin, and PD-L1 in CaSki and SiHa cells transfected with SOX6 expression vector or control vector and WISP3 expression vector or the appropriate control (*n* = 3). **l** Western blot analysis of p53, p21, and PD-L1 in CaSki and SiHa cells transfected with the SOX6 expression vector or control vector in the presence or absence of p53 siRNA (*n* = 3). **m** qRT-PCR analysis of miR-34a expression in CaSki and SiHa cells transfected with the SOX6 expression vector in the presence or absence of p53 siRNA (*n* = 3). **n** Western blot analysis of PD-L1 in CaSki and SiHa cells transfected with the miR-34a mimic or its negative control (*n* = 3). **P < 0.01, ***P < 0.001

SOX6 is a known inhibitor of the Wnt/β-catenin pathway [26]. Thus, we speculated that miR-18a increases PD-L1 levels by modulating the SOX6/Wnt/β-catenin pathway. miR-18a overexpression induced the expression of PD-L1, β-catenin, and STAT3, a downstream effector of the Wnt/β-catenin pathway [15, 16]. Such changes were reversed by treatment with XAV939, a Wnt/β-catenin inhibitor (Supplementary Fig. S8a). XAV939 treatment decreased miR-18a-induced CC cell proliferation and invasion (Supplementary Fig. S8b, c). Forced STAT3 expression upregulated PD-L1 levels in CC cells (Supplementary Fig. S8d). qRT-PCR analysis indicated that miR-18a overexpression led to changes in the expression of Wnt/β-catenin pathway-related genes, including the downregulation of AXIN2 and the upregulation of cyclin D1 and WISP3 (Fig. 4a), suggesting that miR-18a regulates PD-L1 in CC through activation of Wnt/β-catenin/STAT3 signaling.

We next determined whether SOX6 downregulates PD-L1 expression by repressing Wnt/β-catenin pathway activity. Western blotting analysis and TOP/FOP-Flash reporter assays revealed that overexpressing SOX6 inhibited the induction of active β-catenin levels and Wnt/β-catenin activity by miR-18a and downregulated cyclin D1 and PD-L1 induced by miR-18a overexpression in CC cells (Fig. 4b–d). Co-immunoprecipitation assay [29] showed that β-catenin coimmunoprecipitated with SOX6 in nuclear extracts from CC cells (Fig. 4e), demonstrating that SOX6 represses Wnt signaling and PD-L1 expression by binding to β-catenin.

Another possible mechanism by which SOX6 may antagonize Wnt/β-catenin activity is by repressing WISP3, an activator of this pathway [30]. Transient overexpression of SOX6 reduced endogenous WISP3 levels in CC cells (Fig. 4f, g). We identified the SOX6-binding motif within the *WISP3* promoter (Fig. 4h). Chromatin immunoprecipitation (ChIP)-qPCR assays confirmed chromatin occupancy of SOX6 at the promoter regions of *WISP3* and *c-Myc* [27] (Fig. 4i). We co-expressed a SOX6 expression vector with the WT *WISP3* promoter or a version harboring a mutation at the predicted SOX6 binding site in CC cells. Forced SOX6 expression suppressed the activity of the WT, but not the mutant, *WISP3* promoter compared with the controls (Fig. 4j). The inhibition of Wnt/β-catenin activity, PD-L1 expression, and CC cell proliferation and invasion by SOX6 were reversed by ectopic WISP3 expression (Fig. 4k; Supplementary Fig. S9), suggesting that SOX6 increases PD-L1 expression by inhibiting WISP3.

SOX6 is considered an activator of the p53 pathway [27], and p53 suppresses PD-L1 expression via miR-34a [22]. We questioned whether SOX6 regulates PD-L1 expression by elevating p53 and miR-34 levels. Overexpression of SOX6 increased the levels of p53, p21, and miR-34a but decreased PD-L1 expression. These changes were partially reversed by p53 knockdown in CC cells (Fig. 4l, m). Introduction of miR-34a reduced PD-L1 expression and CC cell proliferation and invasion (Fig. 4n; Supplementary Fig. S10). These data reveal that miR-18a increases PD-L1 levels by targeting SOX6 to activate the Wnt/β-catenin pathway and inactivate p53/miR-34a signaling.

### OCT4 transactivates miR-18a in CC cells

Using the TRANSFAC database, we detected the binding sites for the transcriptional factors such as OCT4 and E2F1 [31], and found a positive correlation between OCT4 and miR-18a expression in CC cell lines (Supplementary Fig. S11a, b), indicating that OCT4 might positively regulate miR-18a expression. Transient overexpression of OCT4 increased miR-18a and PD-L1 levels, and siRNA-mediated silencing of OCT4 reduced miR-18a and PD-L1 expression (Supplementary Fig. S11c, d). ChIP-qPCR assays showed the binding of OCT4 to the promoter regions of *miR-18a* and *miR-125b* [32] in CC cells (Supplementary Fig. S11e). The luciferase reporter assays revealed that the luciferase activity of the WT, but not the mutant, *miR-18a* promoter was increased by ectopic OCT4 expression but decreased by OCT4 depletion (Supplementary Fig. S11f). Overexpression of OCT4 increased CC cell proliferation and invasion, but inhibition of miR-18a with an anti-miRNA inhibitor blocked these OCT4-mediated effects (Supplementary Fig. S11g). Knockdown of OCT4 inhibited CC proliferation and invasion, and these suppressive effects were partially rescued by restoring miR-18a levels (Supplementary Fig. S11h). Thus, OCT4 transactivates and upregulates PD-L1 expression, thereby promoting CC cell proliferation and invasion.

### Prognostic impacts of PD-L1, OCT4, WNK2, SOX6, BTG3, and RBSP3 in CC

By analyzing web-based microarray database (GENT, MethHC, and ONCOMINE) that provide gene or miRNA expression patterns across normal and CC tissues, we found that *OCT4*, miR-18a, and *PD-L1* expression levels were significantly upregulated, whereas *WNK2*, *SOX6*, *BTG3*, and *RBSP3* levels were significantly downregulated in CC tissues compared with normal cervical tissues (Supplementary Fig. S12a). Moreover, we evaluated the prognostic impacts of these genes on patient outcomes. Sixty CC patients were classified into two groups using the median expression value as the cutoff. Patients with higher levels of *PD-L1* and *OCT4* or lower levels of *WNK2*, *SOX6*, *BTG3*, and *RBSP3* had much shorter overall survival (Supplementary Fig. S12b). Lastly, we investigated whether elevated miR-18a expression is associated with OCT4 upregulation and with *PTEN*, *WNK2*, *SOX6*, *BTG3*, and *RBSP3* downregulation. qRT-PCR analysis revealed a positive correlation between the expression of miR-18a and *OCT4*, and an inverse correlation of between miR-18a and *PTEN*, *WNK2*, *SOX6*, *BTG3*, or *RBSP3* levels in CC samples (Supplementary Fig. S13c). Altogether, these data support the utilization of PD-L1, OCT4, WNK2, SOX6, BTG3, and RBSP3 levels in clinical outcome predictions of CC.

## Discussion

**Fig. 5:**
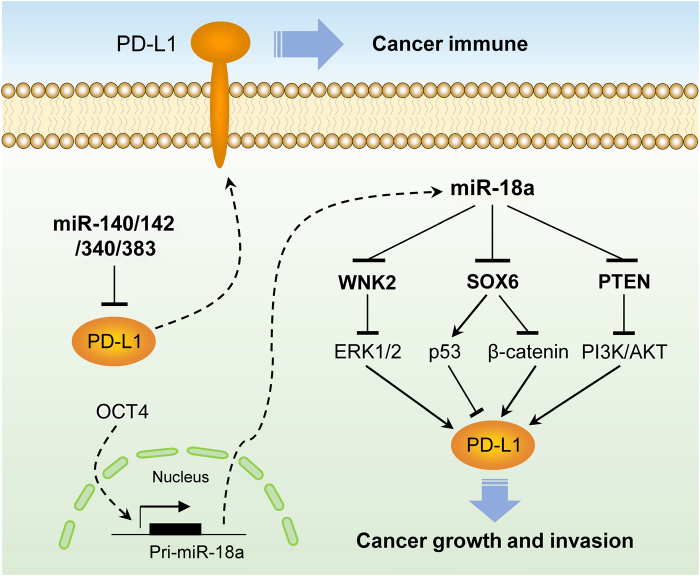
Proposed model for PD-L1 induction in CC. Loss of miR-140/142/340/383 contributes to PD-L1 upregulation in CC. Once miR-18a expression is triggered by OCT4 overexpression, miR-18a enhances PD-L1 levels by targeting PTEN, WNK2 (ERK1/2 pathway inhibitor), and SOX6 (Wnt/β-catenin pathway inhibitor and p53 pathway activator) to activate the PI3K/AKT, MEK/ERK, and Wnt/β-catenin pathways and inhibit the p53 pathway, therefore promoting tumor growth and invasion

The present study shows that PD-L1 functions as a powerful cancer driver by stimulating cancer proliferation and invasion in vitro and facilitating cancer growth in vivo, providing a new insight into the involvement of PD-L1 in CC metastasis. Our results highlighted the crucial roles of miR-140/142/340/383 downregulation and miR-18a upregulation in elevating PD-L1 expression and promoting CC progression. OCT4 induces the transcription of miR-18a, which targets multiple tumor suppressors, enhances PI3K/AKT, MEK/ERK, and Wnt/β-catenin pathway activity, and attenuates p53 signaling, finally leading to PD-L1 upregulation (Fig. 5).

MiRNAs were shown to target PD-L1 [20–22]. We demonstrated that miR-140/142/340/383 are direct suppressors of PD-L1. miR-140/142 were shown to function as tumor suppressors in CC [33, 34]. We also identified two previously uncharacterized miRNAs (miR-340/383) that inhibit CC cell proliferation and invasion by targeting PD-L1.

The PI3K/AKT, MAPK, and Wnt/β-catenin pathways trigger the nuclear translocation of various transcription factors, and binding of the transcription factors such as STAT3 to the *PD-L1* promoter induces the transcription and translation of PD-L1 [11]. We demonstrate that OCT4-activated miR-18a indirectly upregulates PD-L1 by targeting *PTEN*, *WNK2*, and *SOX6* to activate the PI3K/AKT, MEK/ERK, and Wnt/β-catenin pathways and repress p53 signaling. Consistently, dysregulation of PI3K/AKT pathway, MEK/ERK pathway, and p53 pathway represents critical mechanisms of tumor immune escape [35–37].

miR-18a, a member of the miR-17-92 cluster, is frequently overexpressed in human cancers and associated with cancer progression [38]. A previous study reported miR-18a upregulation in CC samples compared with normal cervical samples [39]. We showed here that miR-18a stimulates the metastatic ability of CC cells, and verified therapeutic effectiveness of CRISPR/Cas9-mediated miR-18a disruption in a xenograft mouse model of CC, suggesting that miR-18a-targeted therapy is a potential approach for treating patients with CC. The low miR-18a expression in normal cervical tissues may indicate that miR-18a-targeted therapy will be characterized by high specificity and limited toxicity.

Several mechanisms have been shown to sustain miR-18a expression in cancer cells. Activation of the transcription factors STAT3 and E2F1 induces miR-18a expression through direct binding [30, 40], highlighting the fact that our findings on OCT4 do not represent the only mechanism responsible for miR-18a overexpression in CC. Interestingly, BTG3, a miR-18a target gene identified in this study, can directly bind and inhibit E2F1 [41], indicating the presence of a miR-18a-BTG3-E2F1 feedback loop that leads to persistent miR-18a overexpression in CC cells.

This study uncovers two previously unappreciated mechanisms contributing to PD-L1 overexpression in CC, the miR-140/142/340/383–PD-L1 axis, and the OCT4-miR-18a-PTEN/WNK2/SOX6 oncogenic axis, thereby providing insights into epigenetic/genetic mechanisms underlying the pathogenesis of CC, and highlighting the clinical potential of miRNA-based CC therapy.

## Materials and methods

### Cell culture, reagents, and transient transfection

The CC cell lines (CaSki, ME-180, and SiHa) and pancreatic cancer cell lines (AsPC-1 and PaCa-2) were purchased from the ATCC. The immortalized human endometrial epithelial EM cell line was kindly provided by Satoru Kyo (Shimane University, Japan). Cells were cultured under the recommended conditions. Highly invasive SiHa cells were selected using Matrigel invasion chambers as previously described [24]. These cell lines were routinely tested by PCR for mycoplasma contamination. The MEK inhibitor PD0325901 and the Wnt/beta-catenin inhibitor XAV939 were obtained from Sigma-Aldrich. CC cells were treated with PD0325901 (1 µM) for 48 h or with XAV939 (4 or 8 µM) for 16 h. The miRNA mimics, anti-miRNA inhibitors, siRNAs against PD-L1, p53, and OCT4 and negative control siRNA were obtained from Ambion (Austin, TX). Transient transfection was accomplished with using Lipofectamine 3000 (Invitrogen, Carlsbad, CA) according to the manufacturer’s instructions. The cDNA vectors encoding human PD-L1, SOX6, WNK2, BTG3, RBSP3, WISP3, or OCT4 were from OriGene (Rockville, MD). To generate stable cell lines overexpressing PD-L1, CaSki cells were transfected with a human PD-L1 cDNA expression vector (Origene) using Lipofectamine 3000 (Invitrogen). The respective control vector was used to generate control cell lines. After 24 h, stable transfectants were selected in medium containing 0.5 mg/ml G418 (Sigma-Aldrich). The lentiviral vector harboring shRNA against human PD-L1 and the appropriate control were purchased from the shRNA/ORF Core Facility (MD Anderson Cancer Center). SiHa cells with stable PD-L1 knockdown were generated as previously reported [42]. The expression vectors harboring either full-length or cytoplasmic domain-truncated *PD-L1* gene were constructed and stable cell lines were established as previously reported [43].

### CC specimens and IHC staining

All work related to human tissues was performed under protocols approved by the Institutional Review Board at Sun Yat-Sen University Cancer Center, China. Informed consent was obtained from all patients. Twenty-three paired human CC tissues and adjacent non-tumor tissues were collected at Sun Yat-Sen University Cancer Center. IHC staining was performed using antibodies against PD-L1 (clone E1L3N, Cell Signaling) as previously described [44]. Non-specific IgG was used as a negative control. A semi-quantitative staining score ranging from 0 to 8 was calculated for each specimen by combining the staining intensity (0: negative; 1: low; 2: moderate; and 3: high) and the percentage of positive cells (0: negative; 1+: 0–1%; 2+: 1–10%; 3+: 11–33%; 4+: 34–66%; and 5+: 67–100%). Further, the degree of PD-L1 expression was quantified as follows: Negative expression (0 score), weak expression (1–3 scores), or strong expression (4–8 scores). In addition, a total of 60 pairs of snap-frozen primary CC specimens and adjacent non-tumor cervical tissues were processed for qRT-PCR analysis. Written informed consent was obtained from all patients.

### qRT-PCR analysis of miRNA and mRNAs

Total RNA was isolated using TRIzol (Invitrogen). qRT-PCR analysis of miRNA or mRNA was performed as previously reporetd [45, 46].

### Western blotting

Cell protein extracts were prepared using M-Per Mammalian Protein Extraction Reagent (Pierce, Rockford, IL) according to the manufacturer’s instructions. Total protein was fractionated by SDS-PAGE and transferred onto PVDF membranes. The following primary antibodies were used: PD-L1 (17952-1-AP, ProteinTech), SOX6 (NBP1-85811, Novus), WNK2 (07-2261, Millipore), BTG3 (ab112938, Abcam), RBSP3 (ab106973, Abcam), active β-catenin (05-665, Millipore), OCT4 (ab19857, Abcam). p-AKT (sc-293125), p-ERK1/2 (sc-16982), total β-catenin (sc-7963), ERK1/2 (sc-514302), PTEN (sc-7974), cyclin D1 (sc-8396), p21 (sc-6246), and GAPDH (sc-47724) were obtained from Santa Cruz Biotechnology. GAPDH was analyzed to show equal protein loading. Blots were developed with the enhanced chemiluminescence blotting analysis system (Amersham Pharmacia Biotech, Buckinghamshire, UK). Immunoblot images were digitized and quantified using the ImageJ software. Results were expressed as a relative ratio of PD-L1 to GAPDH and the PD-L1/GAPDH ratio in normal cells was set as 1.

### Cell proliferation assay, colony formation assay, and Matrigel invasion assay

Cell proliferation was determined using the Cell Counting Kit-8 (Dojindo, Kumamoto, Japan) according to the manufacturer’s protocol. In the colony formation assay, 500 cells were added to each well of a 6-well culture plate. After 14 days of culture, cells were fixed using 10% formalin and then stained using 10% Giemsa. Colony formation efficiency was calculated as follows: Plate colony formation efficiency (%) = (number of colonies/number of cells inoculated) × 100. Cell invasion was monitored and analyzed as described previously [45, 46].

### Tumor xenograft experiments

All experiments involving mice were performed in accordance with the guidelines of the Animal Care and Use Committee of Sun Yat-Sen University Cancer Center. Five-week-old female nude mice (BALB/c) were purchased from Shanghai Laboratory Animal Center (Chinese Academy of Sciences, Shanghai, China). SiHa cells (1 × 10^6^) were suspended in phosphate buffered saline (100 µl) and then injected subcutaneously into mice. Tumor size was monitored using digital calipers, and tumor volume was calculated as length × width^2^ x 0.5. All the mice were euthanized at day 35 post-inoculation. The excised tumors were embedded in paraffin and then subjected to IHC analysis of Ki-67 (ab15580, Abcam).

### Microarray experiment

miRNA expression profiling was performed using Superprint G3 Human GE 8x60k Microarrays (Agilent Technologies) as previously described [44]. Microarray expression data were imported into GeneSpring software (Agilent Technologies). Differentially expressed miRNAs and mRNAs (*P* < 0.05 and fold change >2.0) were selected for further analysis. Microarray data was shown in Supplementary Table S1.

### Generation of PD-L1 knockout cells using the CRISPR/Cas9 system

To knockout the endogenous miR-18a gene in SiHa cells, we used CRISPR/Cas9-mediated genome editing as previously described [47]. In brief, two gRNAs designed using CRISPR DESIGN (http://crispr.mit.edu/) to target the human miR-18a genomic sequence were subcloned into the lenti-CRISPR vector, and the inserted gRNAs were verified by DNA sequencing (gRNA-1: 5′-TATGCCAGAAGGAGCACTTA-3′; gRNA-2: 5′-TTATGCCAGAAGGAGCACTT-3′). Stable cell lines were generated by transducing SiHa cells with the lenti-CRISPR-miR-18a vector and selecting transduced cells with 1 μg ml^−1^ puromycin (Sigma-Aldrich). SiHa cells transduced with a lenti-CRISPR-control vector expressing a gRNA targeting GFP were used as the control.

### Luciferase activity assay

The 3′-UTR of *PD-L1* was amplified from human genomic DNA and cloned downstream of the luciferase gene in the pMIR-reporter luciferase vector (Ambion, Austin, TX) as previously reported [48]. To construct the WISP3 or pri-miR-18a promoter luciferase vector, the promoter region encompassing the SOX6 or OCT4 binding site was amplified from human genomic DNA and cloned into the *MluI*/*BglII* sites of the pGL3 vector (Promega). The potential miR-140/142/340/383 binding sites in the 3′-UTR of PD-L1 and the potential SOX6 and OCT4 binding sites in the WISP3 and pri-miR-18a promoter regions were mutated using a QuickChange Site-Directed Mutagenesis Kit (Stratagene, La Jolla, CA). ImiRP was used for mutating miRNA binding sites that are located in the 3′-UTR of target genes [49]. Luciferase activity was measured 48 h after transfection using a Dual Luciferase Assay Kit (Promega).

### TOP-Flash/FOP-Flash reporter assay

The TOP-Flash reporter and pTK-RL plasmids were co-transfected into CC cells (5 × 10^4^) in 24-well plates with the miR-18a mimic and a SOX6 expression vector, and the activity of the Firefly and Renilla luciferase reporters was determined at 48 h after transfection using a Dual Luciferase Assay Kit (Promega). TOP-Flash reporter activity is presented as the relative ratio of Firefly luciferase activity to Renilla luciferase activity.

### ChIP-qPCR assay

Cellular chromatin was immunoprecipitated with an antibody against SOX6 (ab30455, Abcam), OCT4 (ab19857, Abcam), or IgG (control, Santa Cruz Biotechnology) using the Pierce Agarose ChIP Kit (Pierce). The amount of immunoprecipitated DNA was quantified using Takara SYBR Premix Ex Taq II (Takara). The results are presented as the fold enrichment over the IgG control. The human *c-Myc* [27] and *miR-125b* [32] promoters were used as positive controls for SOX6 and OCT4 binding, respectively.

### Statistical analysis

Statistical analyses were performed using SPSS version 22.0 (SPSS Inc., Chicago, IL, USA). All results are expressed as mean ± s.d. All experiments were performed at least three times. Statistical differences between categorical data were evaluated by Fisher’s exact test. Comparisons between groups of continuous variables were performed using Student’s *t*-test (2 groups) or one-way ANOVA test (>2 groups). The Wilcoxon signed-rank test was used to compare two groups of paired nonparametric data. Differences in mRNA or miRNA expression between CC tissues and normal cervical tissues were evaluated using the nonparametric Mann–Whitney U test. Results with two-tailed *P*-values < 0.05 were judged to be statistically significant.

## Electronic supplementary material

Clean Supplementary Figures

Supplementary Table
