# Correction: Control of PD-L1 expression by miR-140/142/340/383 and oncogenic activation of the OCT4–miR-18a pathway in cervical cancer

**DOI:** 10.1038/s41388-019-0677-x

**Published:** 2019-01-24

**Authors:** Peixin Dong, Ying Xiong, Jiehai Yu, Lin Chen, Tang Tao, Song Yi, Sharon J. B. Hanley, Junming Yue, Hidemichi Watari, Noriaki Sakuragi

**Affiliations:** 1 Department of Women’s Health Educational System, Hokkaido University School of Medicine, Hokkaido University, Sapporo, 0608638 Japan; 2 Department of Obstetrics and Gynecology, Hokkaido University School of Medicine, Hokkaido University, Sapporo, 0608638 Japan; 3 Department of Gynecology, State Key Laboratory of Oncology in South China, Sun Yat-Sen University Cancer Center, 510060 Guangzhou, China; 4 Faculty of Medicine, Department of Obstetrics and Gynaecology, The Chinese University of Hong Kong, Shatin, New Territories, Hong Kong China; 5 Department of Pathology and Laboratory Medicine, University of Tennessee Health Science Center, Memphis, TN 38163 USA; 6 Center for Cancer Research, University of Tennessee Health Science Center, Memphis, TN 38163 USA

**Keywords:** Cervical cancer, Immunosurveillance

**Correction to: Oncogene**

10.1038/s41388-018-0347-4; Published online 31 May 2018

In Fig. 2d, the Western blot panels representing GAPDH endogenous loading controls were improperly cropped, leading to four lanes of GAPDH endogenous loading controls for five lanes of PD-L1 protein expressions. The authors apologize for any confusion that this error may have caused. This has now been corrected in both the PDF and HTML versions of the article.

