# Supplementary material for: Control of PD-L1 expression by miR-140/142/340/383 and oncogenic activation of the OCT4–miR-18a pathway in cervical cancer
Source: Oncogene. 2018 May 31;37(39):5257–68. doi: 10.1038/s41388-018-0347-4 (PMC6160397; doi:10.1038/s41388-018-0347-4)

**SUPPLEMENTARY FIGURES**

Control of PD-L1 expression by miR-140/142/340/383 and oncogenic activation of the OCT4–miR-18a pathway in cervical cancer

Peixin Dong, Ying Xiong, Jiehai Yu, Lin Chen, Tang Tao, Song Yi, Sharon JB Hanley, Junming Yue, Hidemichi Watari & Noriaki Sakuragi

**Contents**

**Supplementary Figures**:

**Supplementary Figure S1.**…………….……………..….………………..……….………….……………….**11**

**Supplementary Figure S2.**…………….……………..….………………..……….………….……………….**12**

**Supplementary Figure S3.**…………….……………..….………………..……….………….……………….**13**

**Supplementary Figure S4.**…………….……………..….………………..……….………….……………….**14**

**Supplementary Figure S5.**…………….……………..….………………..……….………….……………….**15**

**Supplementary Figure S6.**…………….……………..….………………..……….………….……………….**16**

**Supplementary Figure S7.**…………….……………..….………………..……….………….……………….**17**

**Supplementary Figure S8.**…………….……………..….………………..……….………….……………….**18**

**Supplementary Figure S9.**…………….……………..….………………..……….………….……………….**19**

**Supplementary Figure S10.**…………….……………..….………………..……….………….………………**20**

**Supplementary Figure S11.**…………….……………..….………………..……….………….………………**21**

**Supplementary Figure S12.**…………….……………..….………………..……….………….………………**22**

**Supplementary Figure Legends**:

**Supplementary Figure S1. identification of miRNAs that directly and indirectly regulate PD-L1 expression.**

(**a**) Western blot analysis and densitometric analysis of PD-L1 expression in the immortalized endometrial epithelial cell line EM (normal), CaSki, ME-180 and SiHa cells (*n* = 3); western blotting for PD-L1 in SiHa cells transduced with PD-L1 shRNA vector or control vector, and in CaSki cells transfected with PD-L1 expression vector or control vector (*n* = 3). (**b**) Western blot analysis of PD-L1 expression in CaSki cells following stable PD-L1 overexpression (left panel). Colony formation and Matrigel invasion assays of CaSki cells with stable PD-L1 overexpression (right panel) (*n* = 3). (**c**) Workflow for identifying miRNAs that directly target PD-L1 (upper panel), and miRNAs that indirectly upregulate PD-L1 expression via targeting PTEN in CC cells (lower panel). (**d**) qRT-PCR analysis of indicated miRNAs in normal, CaSki, ME-180 and SiHa cells, or in normal, AsPC-1 and PaCa-2 cells (*n* = 4). (**e**) qRT-PCR analysis of PD-L1 in SiHa cells transfected with anti-miR-18a inhibitor, indicated miRNA mimics or their negative controls (*n* = 4). (**f**) qRT-PCR analysis of PD-L1 in CaSki cells transfected with miR-18a mimic, indicated anti-miRNA inhibitors or their negative controls (*n* = 4). ^*^*P* < 0.05. ^**^*P* < 0.01. ^***^*P* < 0.001.

**Supplementary Figure S2: Identification of miRNAs that regulate PD-L1 expression.**

(**a**) Morphology of SiHa parental cells and the highly invasive SiHa sublines. (**b**) Heatmap representing miRNAs with > 2.0-fold differential expression between parental and highly invasive SiHa cells. (**c**) Box plot comparing the expression level of the indicated miRNAs in snap-frozen CC tissues (*n* = 60) and adjacent normal cervical tissues (*n* = 60). (**d**) Correlation of PD-L1 expression and that of the indicated miRNAs in 60 snap-frozen CC specimens. (**e**) Kaplan-Meier curves of CC patient survival based on high or low expression of the indicated miRNAs. ****P* < 0.001.

**Supplementary Figure S3. PD-L1 mediates the effects of miR-140/142/340/383 on cervical cancer growth and invasion.**

(**a**) Western blot analysis of PD-L1 expression in SiHa cells transfected with indicated miRNA mimics, together with (or without) PD-L1 expression vector (upper panel) (*n* = 3). Western blot analysis of PD-L1 expression in CaSki cells transfected with indicated anti-miRNA inhibitors, together with (or without) PD-L1 siRNA (lower panel) (*n* = 3). (**b**) Cell proliferation assays and Matrigel invasion assays of SiHa cells transfected with indicated miRNA mimics, together with (or without) PD-L1 expression vector (left panel, *n* = 6), or of CaSki cells transfected as indicated anti-miRNA inhibitors, together with (or without) PD-L1 siRNA (right panel, *n* = 6). ^**^*P* < 0.01.

**Supplementary Figure S4: miR-18a promotes CC cell proliferation and invasion.**

(**a** and **b**) Cell proliferation assays (**a**) and Matrigel invasion assays (**b**) of SiHa, ME-180 and CaSki cells transfected as indicated (*n* = 6). (**c**) Design of sgRNAs for miR-18a. PAM sequences are underlined. (**d**) Structural illustration of the lenti-CRISPR-miR-18a vector. (**e**) Detection of CRISPR/Cas9-mediated DNA cleavage of miR-18a by T7EN1 cleavage assay (*n* = 3). (**f**) DNA sequencing confirming the deletions generated by CRISPR/Cas9 in the *miR-18a* locus. (**g**) qRT-PCR analysis of mature miR-18a expression in SiHa cells transduced with different lenti-CRISPR-miR-18a vectors or control lentiviral vector (left panel, *n* = 3). Immunoblot of the indicated proteins in SiHa cell lines after transduction with lenti-CRISPR-miR-18a vectors or control lentiviral vector (right panel, *n* = 3). (**h, i**) Growth curves (**h**) and Matrigel invasion assays (**i**) of SiHa cells transduced with lenti-CRISPR-miR-18a vectors or control vector (*n* = 8 per group). (**j, k**) Growth curves (**j**) and quantification of tumor weight (**k**) of the tumors that developed in nude mice injected subcutaneously with SiHa cells transduced as indicated (*n* = 8 per group). ^*^*P* < 0.05, ^**^*P* < 0.01 and ^***^*P* < 0.001.

**Supplementary Figure S5. Potential target genes of miR-18a.**

Analysis using algorithms (TargetScan, MIRANDA, DIANA and PITA) predicted that 30 common genes might be potential targets of miR-18a.

**Supplementary Figure S6. Tumor suppressors BTG3 and RBSP3 are direct targets of miR-18a.**

(**a**) Predicted miR-18a binding sites in the 3'-UTRs of *SOX6*, *WNK2*, *BTG3* and *RBSP3*. (**b**) Western blotting analysis of BTG3 and RBSP3 in indicated CC cells and PaCa-2 cells transfected with miR-18a mimic, anti-miR-18a inhibitor or their negative controls (*n* = 3). (**c** and **d**) Luciferase assays for CaSki and ME-180 cells transfected with reporter construct containing the wild-type (WT) or mutant (MU) 3'-UTR of *BTG3* and *RBSP3*, miR-18a mimic or its negative control (**c**), and for SiHa and PaCa-2 cells transfected with reporter construct containing the WT or MU 3'-UTR of *BTG3* and *RBSP3*, anti-miR-18a inhibitor or its negative control (**d**) (*n* = 6). (**e** and **f**) Western blotting analysis of BTG3 and RBSP3 (**e**), cell proliferation assays and Matrigel invasion assays (**f**) for CaSki cells transfected with miR-18a mimic, together with (or without) BTG3 or RBSP3 expression vector (*n* = 3). (**g** and **h**) Western blotting analysis of BTG3 and RBSP3 (**g**), cell proliferation assays and Matrigel invasion assays (**h**) for ME-180 cells transfected with miR-18a mimic, together with (or without) BTG3 or RBSP3 expression vector (*n* = 3). ^***^*P* < 0.001.

**Supplementary Figure S7. miR-18a suppresses the expression of SOX6 and WNK2 to promote cervical cancer cell proliferation and invasion.**

(**a**) Western blotting analysis of SOX6 and WNK2 in ME-180 cells transfected with miR-18a mimic or its negative control, together with (or without) SOX6 or WNK2 expression vector (*n* = 3). (**b, c**) Cell proliferation assays and Matrigel invasion assays of ME-180 cells transfected with miR-18a mimic or its negative control, together with (or without) SOX6 (**b**) or WNK2 (**c**) expression vector (*n* = 4). (**d**) Western blotting analysis of p-ERK1/2, ERK1/2 and PD-L1 in ME-180 cells transfected with miR-18a mimic or its negative control, together with (or without) WNK2 expression vector. (**e**) Western blotting analysis of p-ERK1/2, ERK1/2 and PD-L1 in CaSki and ME-180 cells treated with DMSO or PD0325901 (*n* = 3). (**f**, **g**) Cell proliferation assays and Matrigel invasion assays of CaSki (**f**) and ME-180 (**g**) cells transfected with miR-18a mimic or its negative control, in the presence (or absence) of PD0325901 (*n* = 3). ^***^*P* < 0.001.

**Supplementary Figure S8. The suppression of Wnt/β-Catenin signaling pathway by XAV939 decreased PD-L1 expression and reduced cervical cancer cell proliferation and invasion.**

(**a**) Western blotting analysis of β-Catenin, STAT3 and PD-L1 in CaSki and SiHa cells transfected with miR-18a mimic or its negative control, in the presence (or absence) of XAV939 (*n* = 3). (**b**, **c**) Cell proliferation assays and Matrigel invasion assays of CaSki (**b**) and SiHa (**c**) cells transfected with miR-18a mimic or its negative control, in the presence (or absence) of XAV939 (*n* = 4). (**d**) Western blotting analysis of STAT3 and PD-L1 in CaSki and SiHa cells transfected with STAT3 expression vector or control vector (*n* = 3). ^*^*P* < 0.05. ^**^*P* < 0.01. ^***^*P* < 0.001.

**Supplementary Figure S9. The effects of SOX6 on cervical cancer cell growth and invasion are reversed by ectopic WISP3 expression.**

(**a, b**) Cell proliferation assays (**a**) and Matrigel invasion assays (**b**) of CaSki cells transfected with SOX6 expression vector or control vector, together with (or without) WISP3 expression vector (*n* = 3). (**c**, **d**) Cell proliferation assays (**c**) and Matrigel invasion assays (**d**) of SiHa cells transfected with SOX6 expression vector or control vector, together with (or without) WISP3 expression vector (*n* = 3). ^*^*P* < 0.05. ^**^*P* < 0.01.

**Supplementary Figure S10. miR-34a inhibits proliferation and invasion in cervical cancer cells.**

(**a**, **b**) Cell proliferation assays (**a**) and Matrigel invasion assays (**b**) of CaSki cells transfected with miR-34a mimic or its negative control (*n* = 3). (**c**, **d**) Cell proliferation assays (**a**) and Matrigel invasion assays (**b**) of SiHa cells transfected with miR-34a mimic or its negative control (*n* = 3). ^***^*P* < 0.001.

**Supplementary Figure S11: OCT4 transactivates miR-18a in CC cells**.

(**a**) Schematic showing the predicted OCT4 binding site (shown in red) upstream of the *miR-18a* region. (**b**) Western blot analysis of OCT4 expression in normal cells and CC cell lines (CaSki, ME-180 and SiHa, *n* = 3). (**c**) Western blot analysis of OCT4 in the indicated CC cells transfected with the OCT4 expression vector, OCT4 siRNA or the corresponding negative control (*n* = 3). (**d**) qRT-PCR analysis of OCT4 in the indicated CC cells transfected with the OCT4 expression vector, OCT4 siRNA or the corresponding negative control (*n* = 4). (**e**) OCT4 occupancy at its potential binding site in the *miR-18a* locus was analyzed using ChIP-qPCR assays. miR-125b, a known OCT4 target, was used as a positive control (*n* = 3). (**f**) The indicated CC cells were transfected with a wild-type (WT) or mutant (MUT) pri-miR-18a promoter luciferase reporter vector along with the OCT4 expression vector, OCT4 siRNA or the corresponding negative control. Relative luciferase activity was determined (*n* = 4). (**g**, **h**) Cell proliferation assays and Matrigel invasion assays of CaSki (**g**) and ME-180 (**h**) cells transfected as indicated (*n* = 3). ***P* < 0.01, ****P* < 0.001.

**Supplementary Figure S12: Prognostic impacts of PD-L1, OCT4, WNK2, SOX6, BTG3 and RBSP3 in CC.**

(**a**) Analysis of *PD-L1*, miR-18a, *OCT4*, *WNK2*, *SOX6*, *BTG3* and *RBSP3* expression in CC tissues relative to the corresponding normal cervical tissues (according to the GENT, MethHC and Oncomine databases). (**b**) Prognosis of CC patients stratified by median *PD-L1*, *OCT4*, *WNK2*, *SOX6*, *BTG3* and *RBSP3* mRNA expression (*n* = 30 for the high expression group versus *n* = 30 for the low expression group). (**c**) Correlation of expression between miR-18a expression levels and *OCT4*, *PTEN*, *WNK2*, *SOX6*, *BTG3* and *RBSP3* mRNA expression. ^*^*P* < 0.05, ^**^*P* < 0.01.

**Supplementary Figure S1. identification of miRNAs that directly and indirectly regulate PD-L1 expression.**


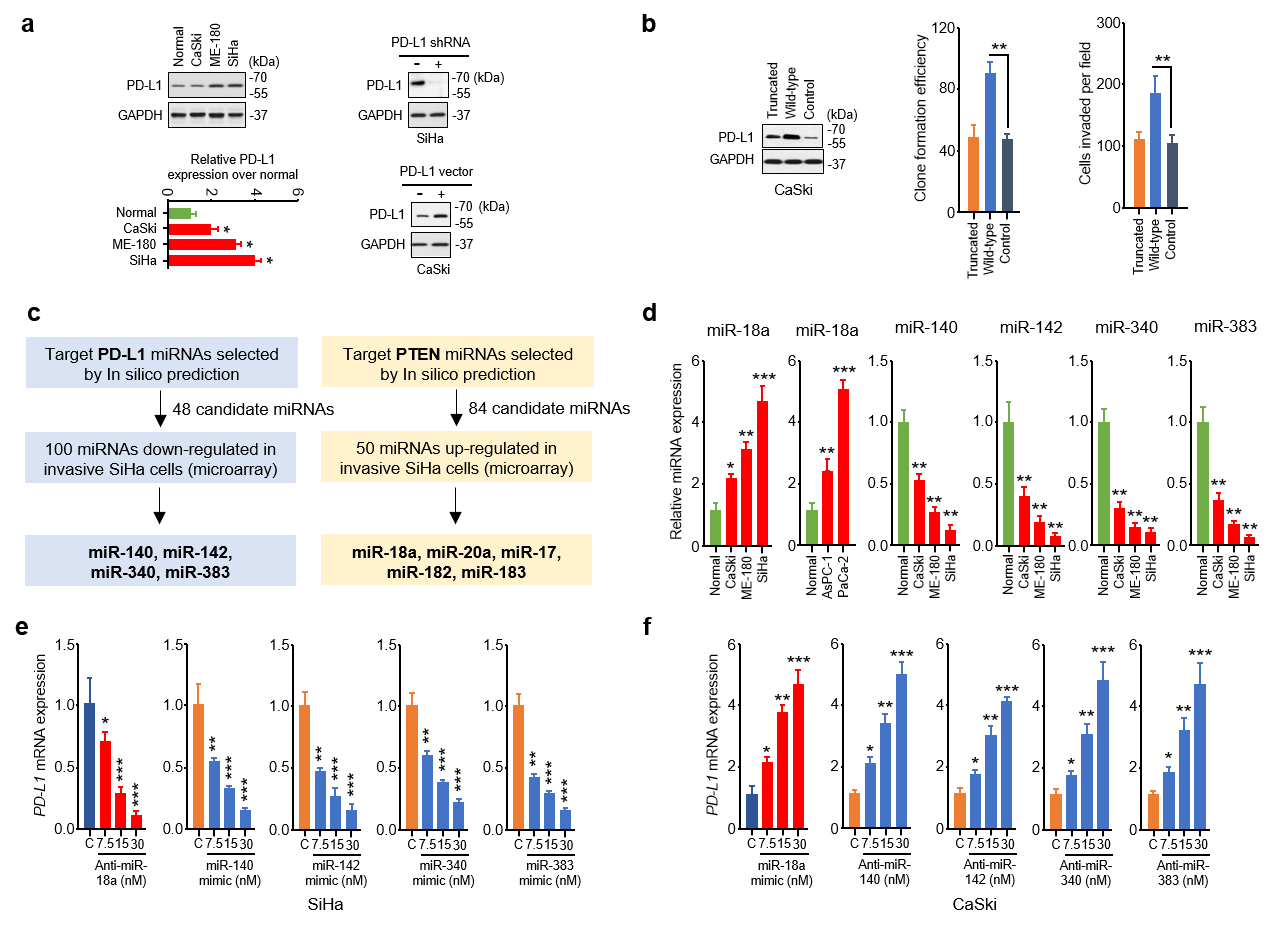


**Supplementary Figure S2: Identification of miRNAs that regulate PD-L1 expression.**


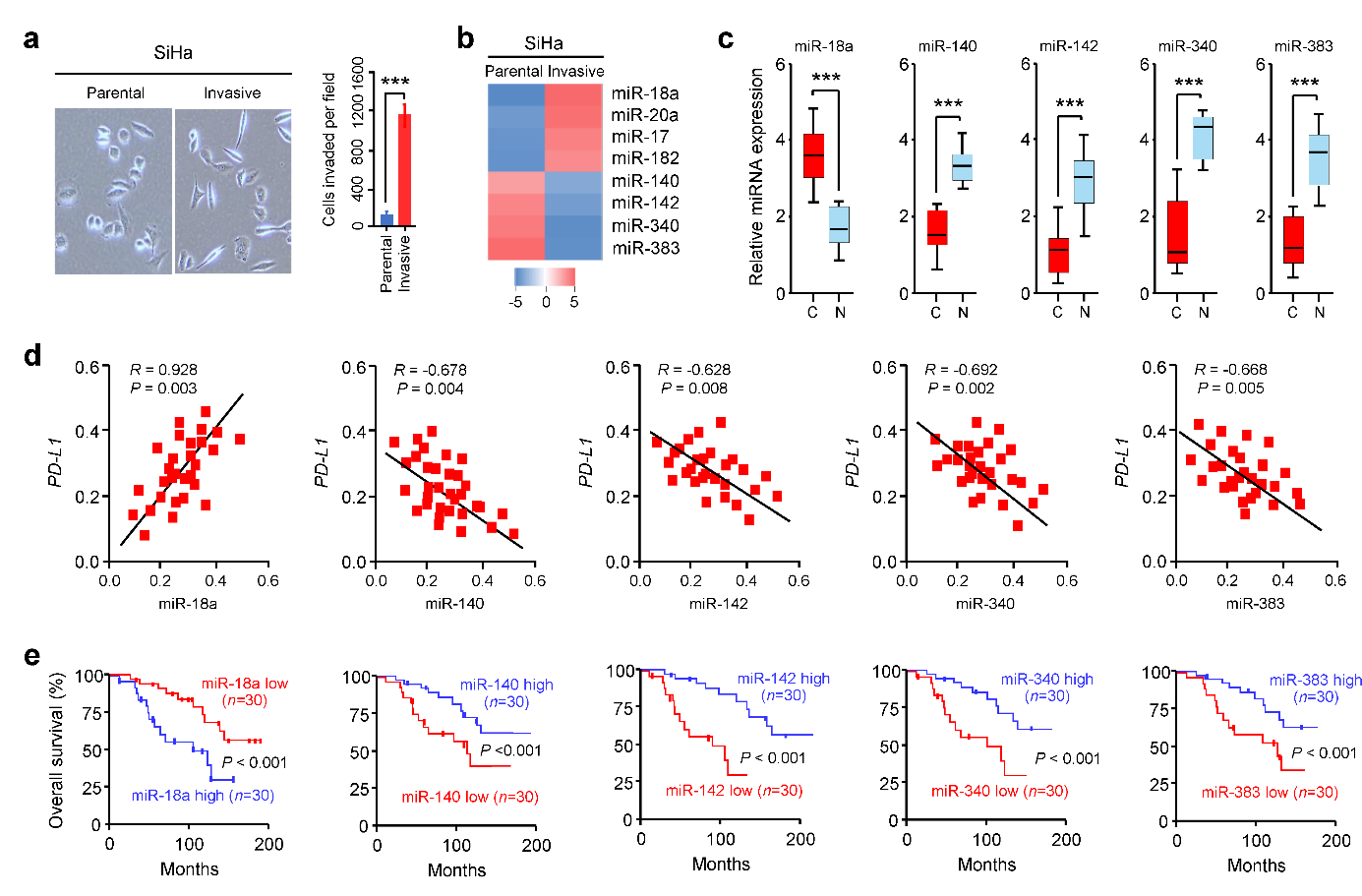


**Supplementary Figure S3. PD-L1 mediates the effects of miR-140/142/340/383 on cervical cancer growth and invasion.**


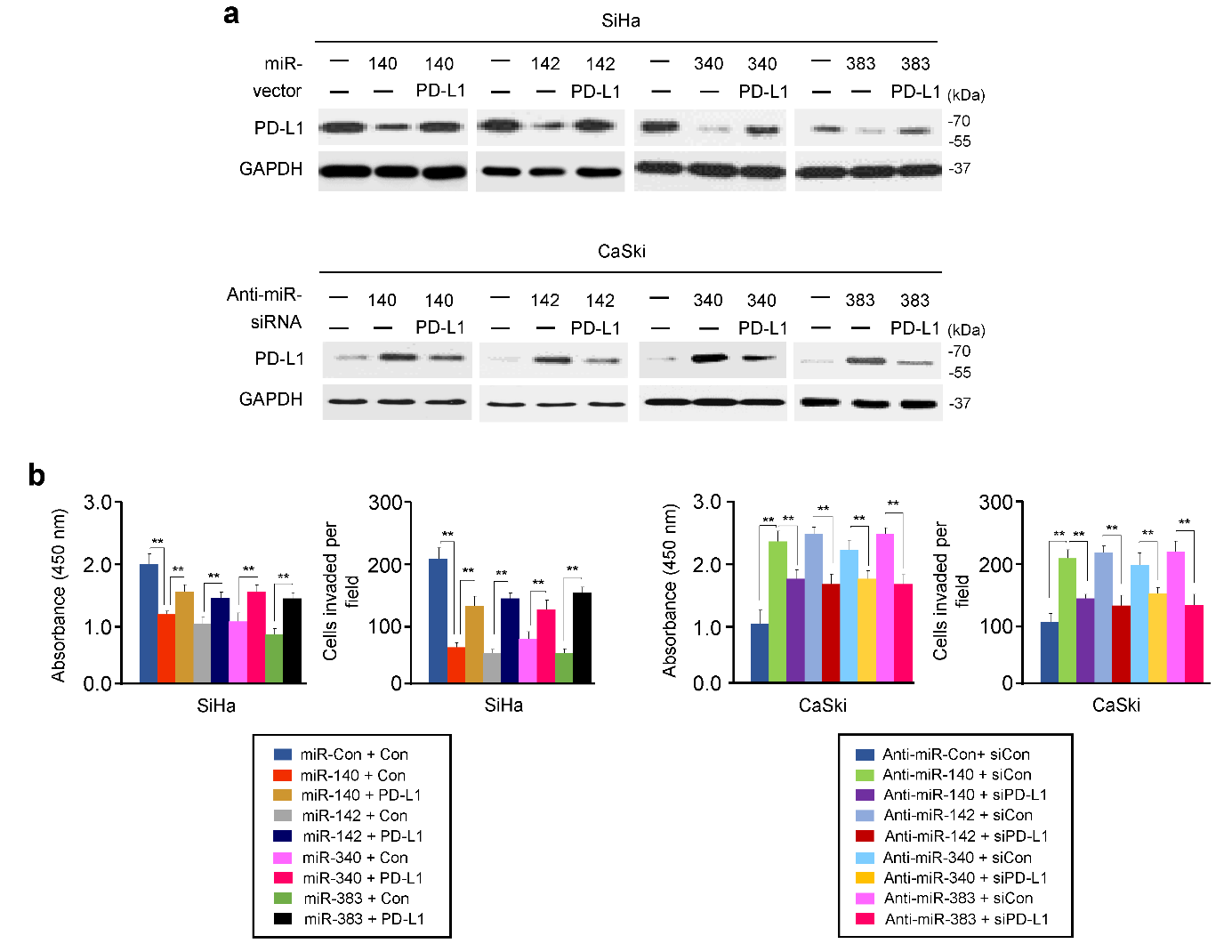


**Supplementary Figure S4: miR-18a promotes CC cell proliferation and invasion.**


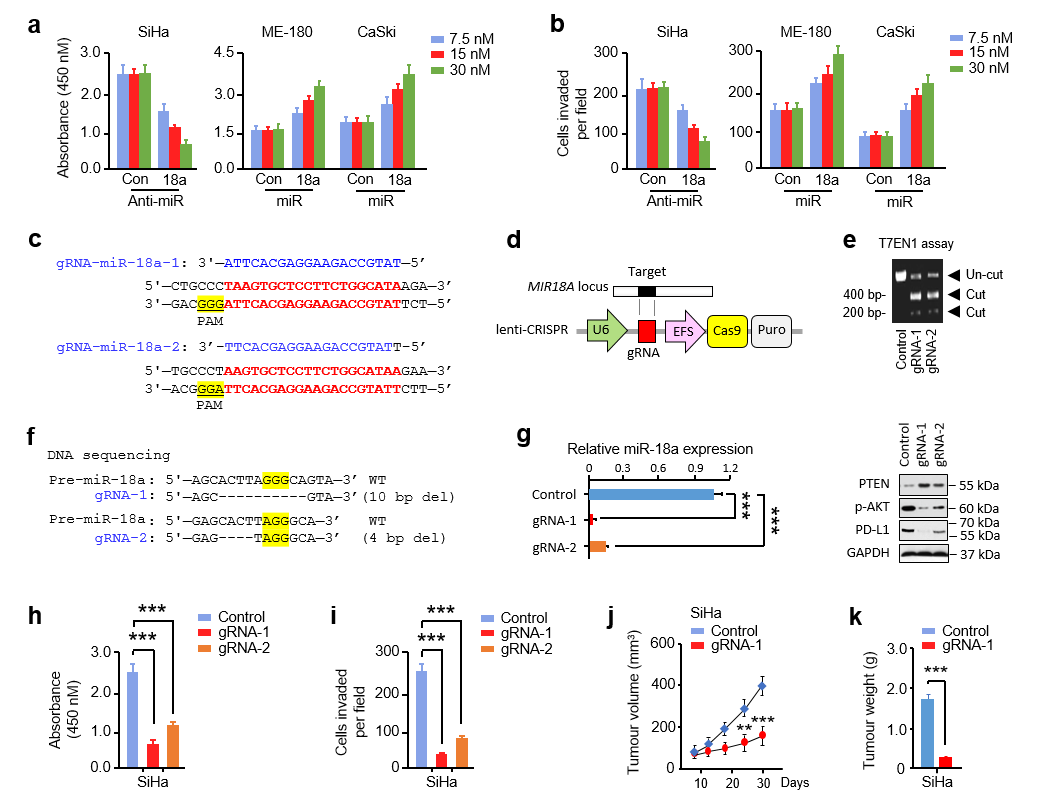


**Supplementary Figure S5. Potential target genes of miR-18a.**


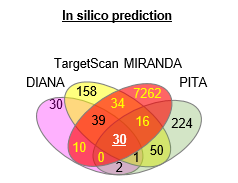


**Supplementary Figure S6. Tumor suppressors BTG3 and RBSP3 are direct targets of miR-18a.**


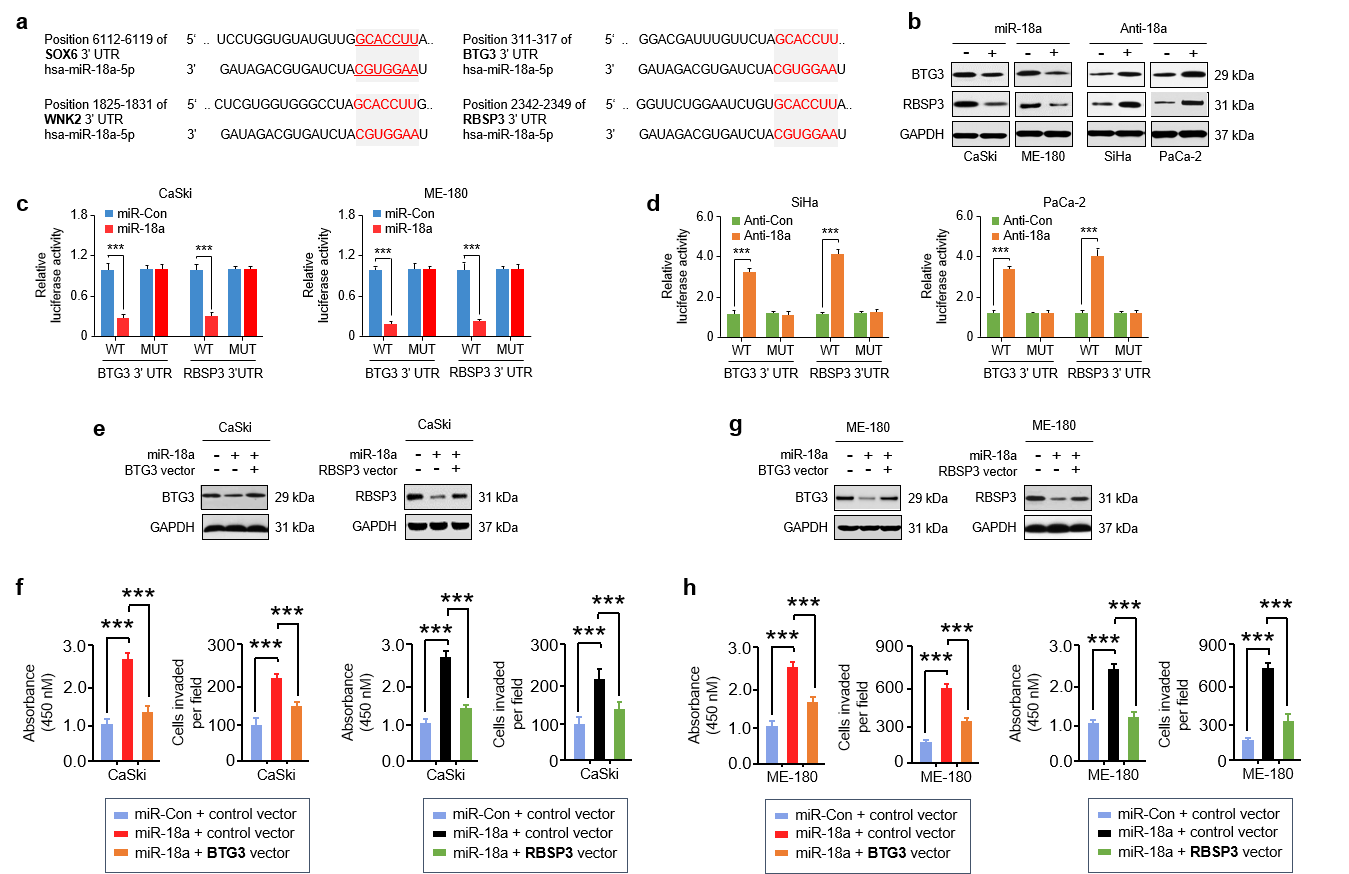


**Supplementary Figure S7. miR-18a suppresses the expression of SOX6 and WNK2 to promote cervical cancer cell proliferation and invasion.**


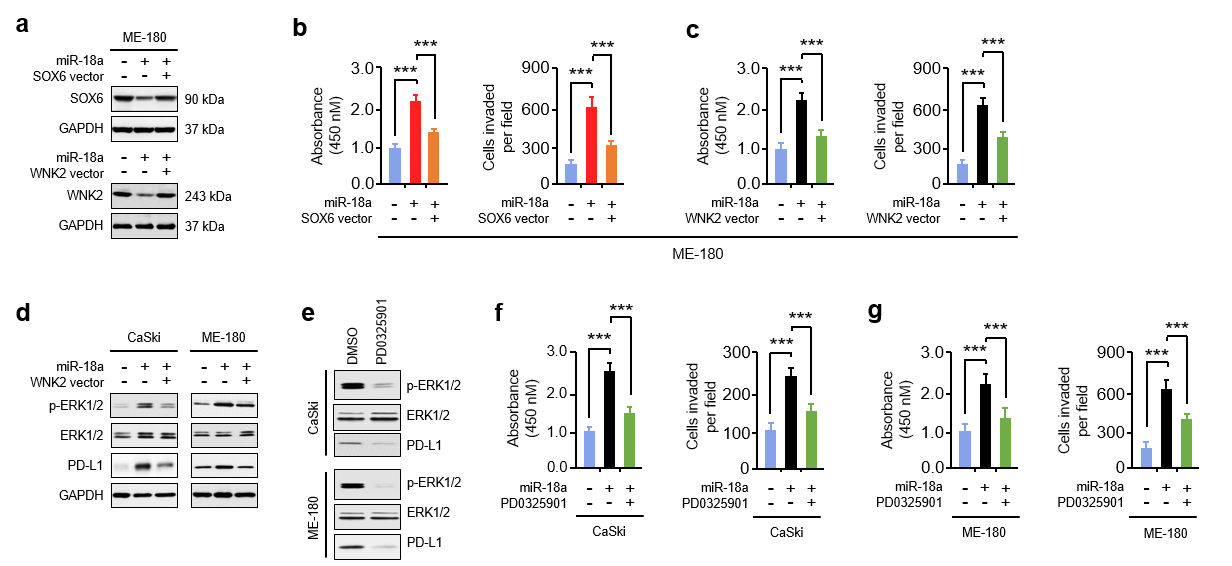


**Supplementary Figure S8. The suppression of Wnt/β-Catenin signaling pathway by XAV939 decreased PD-L1 expression and reduced cervical cancer cell proliferation and invasion.**


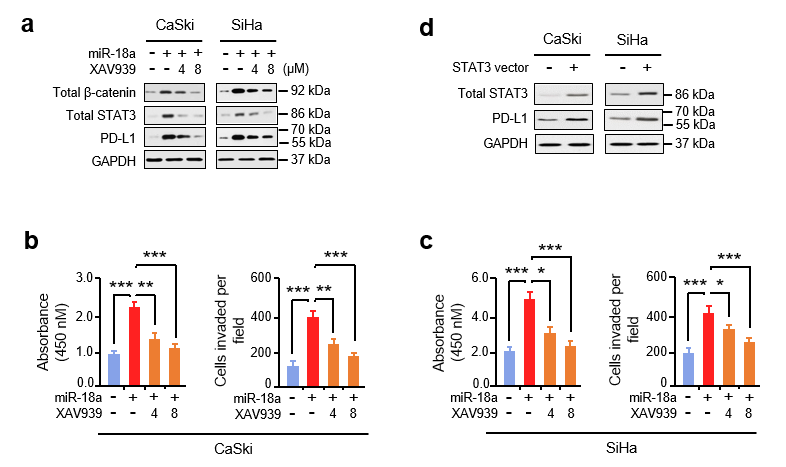


**Supplementary Figure S9. The effects of SOX6 on cervical cancer cell growth and invasion are reversed by ectopic WISP3 expression.**


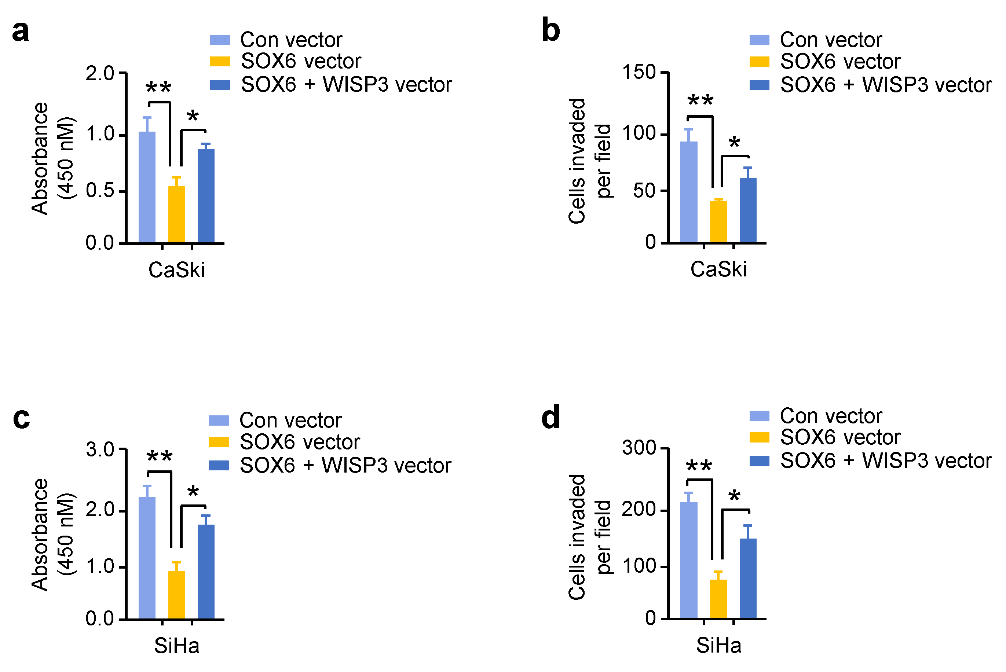


**Supplementary Figure S10. miR-34a inhibits proliferation and invasion in cervical cancer cells.**


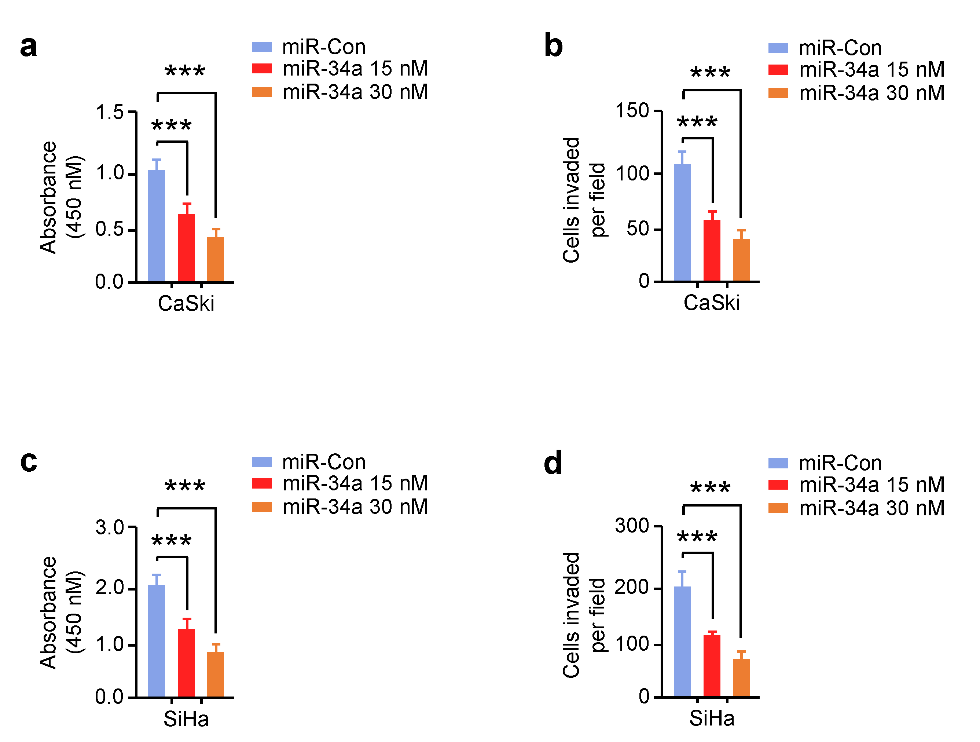


**Supplementary Figure S11: OCT4 transactivates miR-18a in CC cells**.


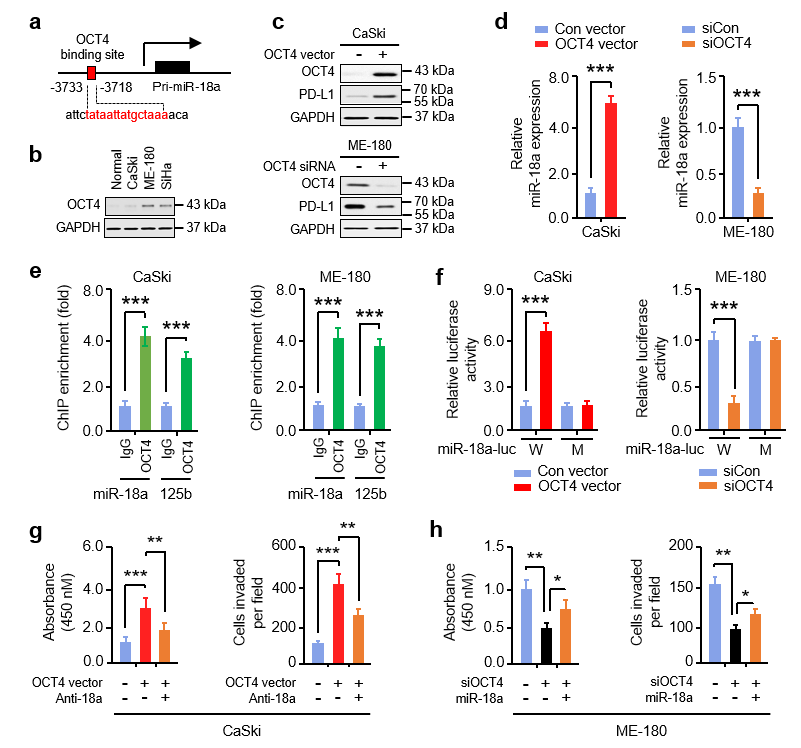


**Supplementary Figure S12: Prognostic impacts of PD-L1, OCT4, WNK2, SOX6, BTG3 and RBSP3 in CC.**


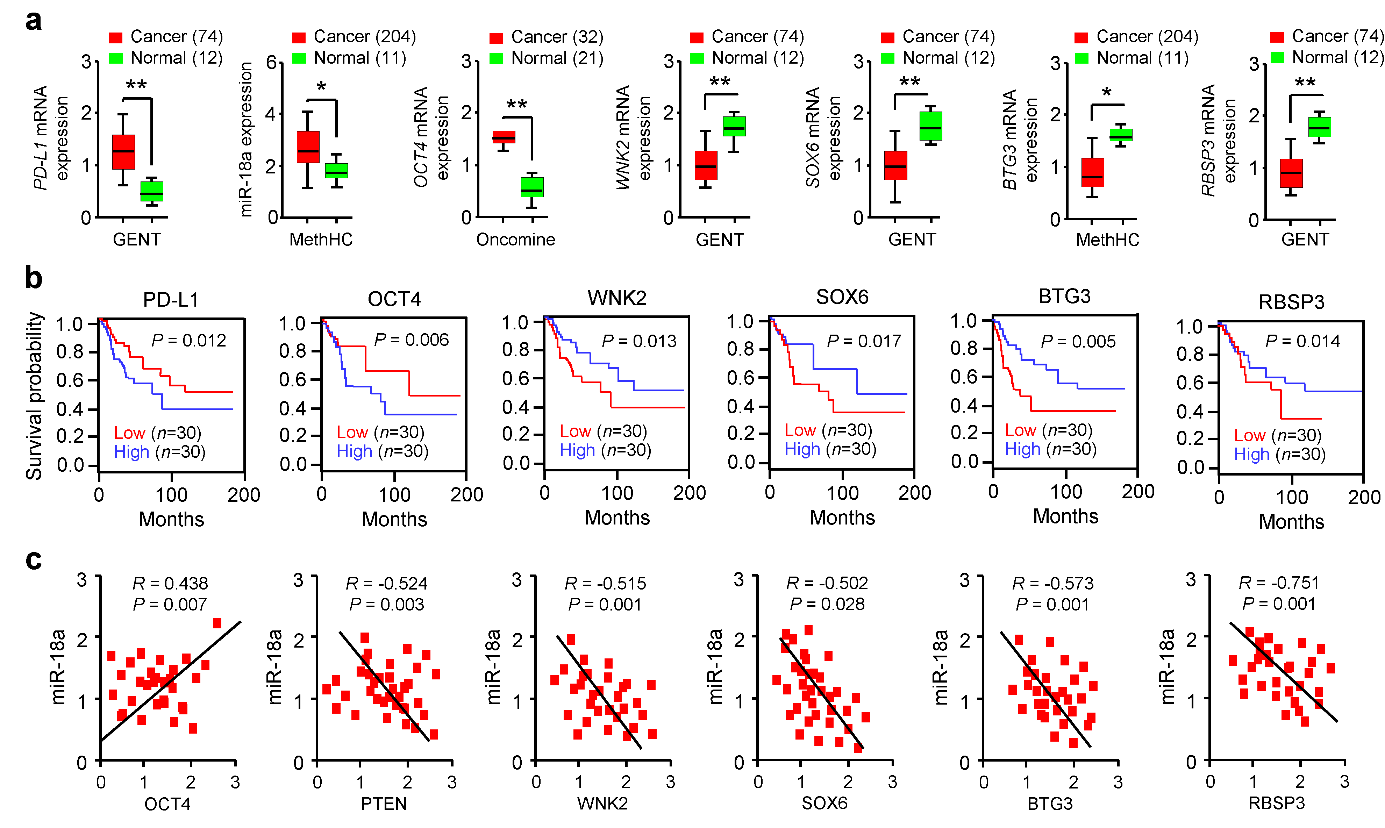

Supplement: Supplementary file 1 — Clean Supplementary Figures [file 41388_2018_347_MOESM1_ESM.docx]
