# Supplementary material for: Control of PD-L1 expression by miR-140/142/340/383 and oncogenic activation of the OCT4–miR-18a pathway in cervical cancer
Source: Oncogene. 2018 May 31;37(39):5257–68. doi: 10.1038/s41388-018-0347-4 (PMC6160397; doi:10.1038/s41388-018-0347-4)
Supplement: Supplementary file 2 — Supplementary Table [file 41388_2018_347_MOESM2_ESM.docx]

**SUPPLEMENTARY TABLE**

Control of PD-L1 expression by miR-140/142/340/383 and oncogenic activation of the OCT4–miR-18a pathway in cervical cancer

Peixin Dong^1,6,7*^, Ying Xiong^2,7^, Jiehai Yu^2^, Lin Chen^2^, Tang Tao^3^, Song Yi^3^, Sharon JB Hanley^1^, Junming Yue^4,5,*^, Hidemichi Watari^6,*^ & Noriaki Sakuragi^1^

**Content**

**Supplementary Table**:

**Supplementary Table S1.**…………….……………..….………………..……….………….……………….**2**

**Supplementary Table S1. miRNAs that were dysregulated in highly invasive SiHa cells.**

| Up-regulated miRNAs | Fold change | *P* value |
| --- | --- | --- |
| hsa-miR-18a | 9.06 | 0.001 |
| hsa-miR-20a | 8.57 | 0.002 |
| hsa-miR-17 | 8.41 | 0.003 |
| Has-miR-182 | 7.64 | 0.001 |

| Down-regulated miRNAs | Fold change | *P* value |
| --- | --- | --- |
| hsa-miR-140 | 0.26 | 0.004 |
| hsa-miR-142 | 0.15 | 0.001 |
| hsa-miR-340 | 0.08 | 0.001 |
| hsa-miR-383 | 0.04 | 0.005 |
